# Impaired CD4^+^ T cell response to apolipoprotein B-100 peptide P210 in atherosclerotic cardiovascular disease is reversed by P210-conjugated nanoparticles

**DOI:** 10.1152/ajpheart.00406.2026

**Published:** 2026-08-21

**Authors:** Paul C. Dimayuga, Jianchang Zhou, Xiaoning Zhao, Janet Wei, Noah Trac, Karla Lambaren, Eun Ji Chung, Bojan Cercek, Prediman K. Shah, Kuang-Yuh Chyu

**Affiliations:** 1 Department of Cardiology, Oppenheimer Atherosclerosis Research Center, Smidt Heart Institute, Cedars-Sinai Medical Center, Los Angeles, California, United States;; 2 Department of Cardiology, Barbra Streisand Women’s Heart Center, Smidt Heart Institute, Los Angeles, California, United States;; 3 Department of Biomedical Sciences, Cedars-Sinai Medical Center, Los Angeles, California, United States;; 4 Department of Biomedical Engineering, University of Southern California, Los Angeles, California, United States

**Keywords:** apoB-100 antigen, atherosclerosis, CD4^+^ T cell, nanoparticle, P210

## Abstract

The apolipoprotein B-100 (ApoB-100) peptide P210 (amino acids 3136–3155) is localized within atherosclerotic plaques, and low IgG autoantibody levels against P210 are associated with a higher risk of plaque rupture and myocardial infarction (MI), suggesting that this humoral immunity is protective in atherosclerotic cardiovascular disease (ASCVD). Although the clinical significance of P210 antibody response has been shown, it remains unclear whether patients with ASCVD also exhibit an impaired T cell response to P210. Thus, we investigated the T cell response to P210 in patients with ASCVD and evaluated its potential as a target for therapeutic intervention. Peripheral blood mononuclear cells (PBMCs) collected from patients with ASCVD and controls were stimulated with the P210 peptide and evaluated for T cell responses using the activated immune markers (AIM) assay. PBMCs from patients with ASCVD stimulated with P210 had a significantly impaired CD4^+^CD69^+^CD154^+^ T cell response, which persisted into follow-up a year later. Significantly reduced CD4^+^ T follicular helper cell responses to P210 were also noted in patients with ASCVD. Given the beneficial immunomodulation by P210 nanoparticles (P210-PAM) that resulted in reduced atherosclerosis in mice, we investigated its effect on PBMCs from patients with ASCVD. Treatment with P210-PAM significantly improved the impaired CD4^+^CD69^+^CD154^+^ and CD4^+^CD134^+^CD137^+^ T cell activation in response to P210. The study suggests potential future clinical translation for P210-PAM as an immune-modulation strategy for ASCVD.

## NEW & NOTEWORTHY

Patients with ASCVD have reduced levels of IgG reactive with the apoB-100 peptide P210. Although suggestive of immune suppression specific to P210, it is unclear whether this involves T cell responses as well. The current report demonstrates reduced T cell response to P210 in patients with ASCVD that is reversed by P210-conjugated nanoparticles.

## INTRODUCTION

Accumulating evidence supports the role of adaptive immunity in the inflammatory cascade in atherosclerotic cardiovascular disease (ASCVD) (1–7). Analysis of inflammatory cells in atherosclerotic plaques and peripheral blood has demonstrated the presence of antigen-activated T cells (8–11). Given that antigen specificity is fundamental to adaptive immune responses, it offers an opportunity for selective pathway modulation. Investigations focusing on antigen-specific immune modulation could lay the groundwork for novel therapies that target pathways with greater precision and efficacy.

There is increasing evidence supporting the inextricable link between altered immune responses to the apolipoprotein apoB-100 (apoB-100) on low-density lipoprotein (LDL)-cholesterol (LDL-C) and ASCVD (1–7). ApoB-100 peptide antigens, such as P210 (aa 3136–3155), are found in atherosclerotic plaques (12), and antibodies reactive to these apoB-100 peptides are found in patient plasma (5, 6). Low P210 IgG levels are associated with a higher risk of plaque rupture and myocardial infarction (MI) (6, 7). However, the nature of the intrinsic T cell response to P210 in both healthy subjects and patients with ASCVD remains unknown. Also unknown is the intrinsic response to P210 by T follicular (Tf) cells that support antigen-specific B cells. The expression of C-X-C motif chemokine receptor 5 (CXCR5) on recently activated T cells identifies antigen-specific Tf cells with cluster of differentiation (CD)4^+^ Tf helper (Tfh) cells that provide B cell help and CD8^+^ Tf-like cells that regulate B cell function (13–15).

We have previously demonstrated that P210, formulated either as a conjugated epitope with an adjuvant or as self-assembling peptide amphiphile micelles (P210-PAMs), conferred atheroprotection in murine models via modulating T cell responses and macrophage phenotypes (16, 17). Building on these findings, the present study aimed to characterize the T cell response to P210 in human cohorts. We analyzed specific T cell profiles in patients with stable ASCVD and acute coronary syndrome (ACS) following ex vivo P210 stimulation. We also followed a subgroup of patients for a year to test the persistence of the T cell profiles in response to P210. Finally, given the clinical application of antigen-specific immune modulation in other immune-mediated disorders (18) and the efficacy of P210-PAM in mitigating atherosclerosis in mice (17), we evaluated the therapeutic potential of P210-PAM using patient-derived PBMCs.

## MATERIALS AND METHODS

### Human PBMC

Peripheral blood samples were collected at enrollment for PBMC isolation consented under the Institutional Review Board (IRB) protocols Pro00047460 and Pro00058160 approved by the Cedars-Sinai Institutional Review Board. All subjects in the studies gave written informed consent for the use of nonidentifiable data. The study protocol conforms to the ethical guidelines of the 1975 Declaration of Helsinki.

Patients with ACS (*n* = 11) admitted to the Cardiac Intensive Care Unit at Cedars-Sinai Medical Center were enrolled with the following exclusions: inability to give informed consent, age less than 18 yr old, active cancer treated with chemotherapy or radiation, patients taking immunosuppressive drugs, and pregnant women.

ASCVD patient cohorts with a diagnosis of peripheral artery disease (PAD, *n* = 6) or history of prior MI (*n* = 14) or stroke (*n* = 13) 3–12 mo postevent were also enrolled. Inclusion criteria for non-ACS ASCVD patients were recent MI (defined by evidence of myocardial necrosis in a clinical setting consistent with acute myocardial ischemia); recent ischemic stroke (defined as imaging evidence of focal cerebral ischemic injury in a defined vascular distribution with symptoms of neurological dysfunction persisting >24 h); recent diagnosis of symptomatic lower extremity PAD (defined as onset of symptomatic lower extremity intermittent claudication and ankle-brachial index ≤ 0.9, or critical limb ischemia); and subjects with recent or planned prescription of statin and antiplatelet therapy as recommended by the American Heart Association guidelines, within 6 mo of recruitment. Exclusion criteria included age <18 yr; contraindications to MRI, including magnetically activated implanted devices (such as internal cardiac defibrillator or insulin pumps) or untreatable claustrophobia; subjects with a history of four or more prior gadolinium contrast scans; pregnancy; body mass index (BMI) > 40; current high-intensity statin use greater than 6 mo; active unstable cardiac conditions such as acute coronary syndrome, decompensated heart failure, or uncontrolled arrhythmia; life expectancy <5 yr; creatinine clearance <45 or allergy to gadolinium contrast; severe allergy to animal dander; and additional exclusion for stroke was atrial fibrillation, other cardiac sources of embolism, subarachnoid or cerebral hemorrhage, or cerebral venous thrombosis. Patient characteristics are given in Table 1.

PBMCs were isolated using Ficoll (GE Healthcare) density gradient centrifugation and cryopreserved in a commercially available cryogenic solution (Immunospot) and stored in liquid nitrogen (17, 19, 20). Healthy control PBMCs were isolated from remnant blood in Leukocyte Reduction System cones from healthy platelet donors (*n* = 16) from the Cedars-Sinai blood bank.

### P210-PAM Preparation

P210-PAMs were prepared as previously described (17). A 1′−3′ dihexadecyl-*N*-succinyl-l-glutamate (diC_16_) hydrophobic lipid tail was synthesized by mixing hexadecanol, l-glutamic acid, and para-toluenesulfonic acid, yielding 1′−3′-dihexadecyl l-glutamate, and purified through Buchner funnel filtration with acetone. Succinic anhydride was added in tetrahydrofuran and chloroform mixture to yield the crude diC_16_ product, which was crystalized overnight at 4°C and further purified with diethyl ether by Buchner funnel filtration.

Following diC_16_ synthesis, 1 mmol of P210 (KTTKQSFDLSVKAQYKKNKH) peptide was synthesized using an automated PS3 peptide synthesizer (Gyros Protein Technologies, Tucson, AZ) via 9-fluorenylmethoxycarbonyl-mediated solid phase peptide synthesis. P210 peptide was conjugated to 1 mmol of diC_16_ overnight by shaking with *N,N*-diisopropylethylamine (DIPEA) and *O*-benzotriazole-*N,N,N’,N’*-tetramethyl-uronium-hexafluoro-phosphate (HCTU). The P210-diC_16_ conjugate was cleaved from resin using trifluoroacetic acid, precipitated with ice-cold ether, and lyophilized. Peptide amphiphiles were purified by reverse-phase high-performance liquid chromatography, and P210-PAMs were self-assembled through thin-film hydration (17).

### Activation-Induced Marker Assay of Human PBMCs

T cell activation-induced markers (AIMs) were tested after PBMC stimulation with P210 peptide for 18 h (17, 20, 21). Cryopreserved PBMCs were thawed, rinsed in antiaggregation solution (Immunospot), and seeded in culture plates at a density of 3 × 10^6^ cells/mL in Roswell Park Memorial Institute 1640 medium (RPMI 1640 medium, Thermo Fisher Scientific) supplemented with 10% heat-inactivated pooled human serum (Innovative Research, Inc) and 1X antibiotic/antimycotic (Gibco). After resting for 4 h, cells were preincubated with 0.5 lg/mL anti-CD40 antibody for 15 min, then stimulated with 20 μg/mL P210 peptide or P210-PAM, whereas cells without treatment served as nonstimulated control. A previously reported, type VI collagen a1 (Col6A1) peptide was used as unrelated stimulation (22). Cells were harvested 18 h after seeding, stained for viability (LIVE/DEAD Fixable Aqua Dead Stain Kit, Thermo Fisher Scientific), and subjected to cell surface staining for flow cytometric analysis (BD LSRFortessa Cell Analyzer) using antibodies: CD4, CD8, CD25, CD69, OX40 (CD134), CD137 (4-1 BB), and CD154 (CD40L). Isotypes were used as staining controls, and CD14, CD16, and CD19 antibodies were used as dump staining within the nonviable cell gate to exclude B cells, dendritic cells (DCs), macrophages, granulocytes, eosinophil cells, and neutrophil cells. For T follicular cell assay, the antibody for CD69 was replaced with CXCR5. The results are expressed as fold change (the ratio between the signal in the antigen-stimulated and the signal in the unstimulated condition) for each sample, with group means at a twofold change considered as the activation threshold (17, 20, 21). Antibody clones and sources are listed in APPENDIX Table A1.

### Statistics

Data presented as means ± standard deviation (SD) or displayed as dot plots. D’Agostino–Pearson test was used to determine normality of data. Statistical analysis used unpaired or paired *t*-test for normally distributed data; *F* test was used to compare variances for Welch’s correction; otherwise, Mann–Whitney *U* test to compare independent data or Wilcoxon signed-rank test to compare paired data was used. Categorical data are presented as counts (percentage). *P* value less than 0.05 was considered significant. GraphPad Prism 10.0.0 was used for statistical analysis.

## RESULTS

### ASCVD T Cell Profile in Response to P210

Activation-induced marker (AIM) assay (17) was used to evaluate T cell phenotypes in response to P210 stimulation in controls (*n* = 16) and patients with ASCVD (*n* = 44). Common markers utilized in the AIM assay are CD69, CD154, CD134, and CD137. CD69 is an early marker of T cell activation, and CD154 is expressed transiently after T cell receptor stimulation to provide help to B cells and antigen-presenting cells (21). CD134 is upregulated on T cell activation involved in T cell expansion (23), whereas CD137 promotes T cell survival during antigen-specific T cell responses (24). Given that nonresponsive cell markers would have a ratio of 1, markers that showed mean activation at or near a twofold increase (17, 21) in either group were selected for group comparison.

The gating scheme for activation markers is depicted in Fig. 1A and APPENDIX Fig. A1. CD4^+^CD69^+^CD154^+^ T cells were significantly reduced in PBMCs of patients with ASCVD (*n* = 37) compared with healthy controls (*n* = 16), which showed a twofold increase (Fig. 1B, *P* = 0.02). In contrast, CD4^+^CD134^+^CD137^+^ T cell (Fig. 1C; ASCVD *n* = 43, control *n* = 16) and CD8^+^CD134^+^ T cell (Fig. 1D; ASCVD *n* = 39, control *n* = 11) responses were not different between the groups (*P* = NS). The results suggest that P210 specifically provokes differential CD4^+^CD69^+^CD154^+^ T cell responses between controls and patients with ASCVD. To verify the specificity of the response to P210, a peptide derived from human Col6A1 that was reported as an atherosclerosis antigen (22) was tested as a non-apoB-100 antigen stimulus in the AIM assay. Col6A1 peptide stimulation did not provoke CD4^+^CD69^+^CD154^+^ T cell response and was not different between control (*n* = 14) and ASCVD (*n* = 24; Fig. 1E, *P* = NS). There was no significant difference in age between control (53.0 ± 11.1 yr) and patients with ASCVD (59.0 ± 11.8 yr; *P* = NS).

### Persistence of T Cell Response to P210 in ASCVD

To evaluate whether the T cell profiles in patients with ASCVD persisted over time, we performed longitudinal profiling on PBMCs from a subgroup of patients with prior MI or stroke, re-sampled approximately one year after baseline (*n* = 15). T cell response to P210 at follow-up remained unchanged compared with baseline (Fig. 1, F–H, *P* = NS). The results indicate that T cell profile in response to P210 is persistent and retains an immune-suppressed phenotype in patients with ASCVD.

### Reduced Tf Cell Response to P210 in ASCVD

The suppressed CD4^+^ T cell profile in patients with ASCVD compared with healthy subjects suggested that CD4^+^ T cell response to P210 is impaired in patients. Combined with the reports of increased risk of MI in patients with reduced P210 IgG levels (5–7), the results suggest a potential association between the cellular and humoral adaptive immune response to P210 in MI. Given that CD4^+^CD154^+^ T follicular helper (Tfh) cells expressing CXCR5 are essential for antigen-specific B cell antibody production (25, 26), Tfh cell response to P210 was evaluated in patients with ACS to assure all tested samples were from confirmed patients with MI to align with previous reports (5–7).

The gating scheme for Tf analysis is depicted in Fig. 2A. CD4^+^CD137^+^CXCR5^+^ (Fig. 2B, *P* = 0.0006) and CD4^+^CD154^+^CXCR5^+^ (Fig. 2C, *P* = 0.02) Tfh cells were significantly reduced by P210 stimulation of PBMCs from patients with ACS (*n* = 11). CD8^+^CD137^+^CXCR5^+^ (Fig. 2E, *P* = 0.03) and CD8^+^CD134^+^CXCR5^+^ (Fig. 2G, *P* = 0.02) Tf-like cells were also significantly reduced by P210 stimulation. No differences in CD4^+^CD134^+^CXCR5^+^ Tfh cells (Fig. 2D, *P* = NS) and CD8^+^CD154^+^CXCR5^+^ Tf-like cells (Fig. 2F, *P* = NS) were observed.

### P210-PAM Nanoparticles Reversed the Suppressed CD4^+^ T Cell Response to P210

The results thus far suggest that patients with ASCVD exhibited an impaired CD4^+^ T cell response to P210, and to a lesser extent, CD8^+^ T cell response. Thus, modulating this impaired CD4^+^ T cell response may be a potential therapeutic target in ASCVD. We previously reported that linking P210 to amphiphilic micelle nanoparticles (P210-PAM) as an immune modulation therapy reduced experimental atherosclerosis in a mouse model (17). To extend the translational relevance of these findings, the effect of P210-PAM on T cell profiles was tested on PBMCs of control subjects and patients with ASCVD. Due to cell number constraints following prior assays, analysis was limited to subjects with sufficient remaining cells.

Compared with unconjugated P210 stimulation, P210-PAM had no significant effect on CD4^+^CD69^+^CD154^+^ T cells in PBMCs from control subjects (Fig. 3A, *P* = NS; *n* = 12). On the other hand, CD4^+^CD69^+^CD154^+^ T cell response in patients with ASCVD was significantly increased to activation threshold by P210-PAM compared with unconjugated P210 stimulation (Fig. 3B, *P* = 0.006; *n* = 16).

There was no significant difference in CD4^+^CD134^+^CD137^+^ T cells in control subjects (Fig. 3C, *P* = NS; *n* = 13) but was significantly increased in patients with ASCVD (Fig. 3D, *P* = 0.003; *n* = 24) in response to P210-PAM compared with unconjugated P210. No differences were observed in CD8^+^CD134+ T cells between P210 and P210-PAM stimulation of control subjects (Fig. 3E, *P* = NS; *n* = 9) or ASCVD (Fig. 3F, *P* = NS; *n* = 20).

## DISCUSSION

The study expands on prior work supporting the potential role of the adaptive immune response to the ApoB-100 peptide P210 in ASCVD. The results show that patients with ASCVD have impaired CD4^+^ T cell response to P210 that persists over time and includes CD4^+^ Tf cells. P210 linked to nanoparticles partially reversed the impaired CD4^+^ T cell response to P210 implying therapeutic potential.

CD4^+^CD69^+^CD154^+^ T cells are a subset of activated antigen-responsive CD4^+^ T cells that indicate T cell activation with enhanced helper capacity. The reduced CD4^+^CD69^+^CD154^+^ T cell response to P210 observed in patients with ASCVD, coupled with previously reported low P210 IgG levels in patients at higher risk for MI (5–7), suggests an impairment of both the humoral and cellular arms of the adaptive immune response to P210.

Among potential mediators of IgG generation and maintenance are Tf cells that support antigen-specific B cells. Considering this function of Tf cells, we characterized the Tf cell profile in response to P210 in patients with ACS, assuring that the patient cohort tested for Tf cell profile was from patients with established atherosclerosis. The expression of activation markers CD134 (OX40), CD137 (4-1BB), and CD154 (CD40L) on CD4^+^CXCR5^+^ or CD8^+^CXCR5^+^ T cells identifies recently activated, antigen-specific follicular T cell populations with CD4^+^ Tf cells providing B cell help and CD8^+^ Tf-like cells with B cell helper function. Reduced CD4^+^CD154^+^CXCR5^+^ and CD4^+^CD137^+^CXCR5^+^ T cells after P210 stimulation is consistent with impaired Tf cell response in ACS. CD154 is the CD40 ligand, which is required for antibody production by antigen-specific B cells (25), whereas CD137 is necessary for the generation and maintenance of memory Tf cells (26). Thus, reduced CD40 signaling and CD137-mediated memory Tf cell maintenance in response to P210 is consistent with low P210 IgG levels in patients at risk of MI (5–7).

Reduced P210 IgG levels are associated with increased risk for an acute cardiac event (6, 7). Imai et al. (7) showed that low P210 IgG levels are associated with unstable plaque characteristics and may be a marker of unstable coronary artery plaques that are associated with higher risk of rupture and thrombosis. The role of reduced P210 IgG in the pathophysiology of human ASCVD has not yet been clarified, but induction of P210 IgG response and passive administration of recombinant P210 IgG into apoE^−/−^ mice reduced atherosclerosis (27), suggesting a beneficial role.

Antigen-specific immune modulation as therapy for ASCVD has increasingly gained interest from research laboratories (28–30), including ours (31). To this end, we have developed a nanoparticle approach for modulating the immune response to P210 by generating amphiphilic micelles conjugated to the peptide (P210-PAM) that reduced atherosclerosis in a mouse model (17). PAMs enhance antigen delivery by protecting peptides from degradation and clearance and also providing a scaffold for increased peptide density for antigen presentation (32, 33). Consistent with this mechanism, we previously demonstrated uptake of P210-PAM by murine DCs and observed costaining of P210-PAM with MHC-I on the DC surface (17). Solid-core nanoparticles such as cylindrical micelles with antigen on the surface like P210-PAM (17) can promote robust T cell and humoral responses (34, 35). Among other crucial characteristics of PAMs are increased uptake and self-adjuvanting properties (36). Consistent with our prior report (17), P210 on superparamagnetic iron-oxide nanoparticles has also been shown to elicit CD4+ and CD8+ T cell responses and humoral immunity against P210 (37).

In the current study, the impaired CD4^+^ T cell response to P210 persisted to the 1-year follow-up, suggesting that the immune phenotype associated with the disease may be a target for immune modulation therapy. In support of this, P210-PAM altered the intrinsic response of ASCVD T cells to P210 compared with unconjugated peptide, resulting in significantly increased CD4^+^CD69^+^CD154^+^ and CD4^+^CD134^+^CD137^+^ T cell responses that were not observed in the control. The results suggest that the impaired immune response to P210 in ASCVD can be potentially reversed and strengthens the rationale for the development of an approach targeting P210 for clinical translation.

Our study has several limitations. The limited number of PBMCs retrieved from patients constrained our ability to test all assays in all subjects. Lack of longitudinal follow-up samples in all patients prevented us from evaluating potential time-dependent changes in T cell response. In vitro assays to evaluate T cell responses may not completely recapitulate the complex physiology of human immune activation, thus limiting direct application and interpretation of our findings in vivo. The possible differences in statin use between patients with ASCVD and healthy controls may have contributed to the observed T cell response. However, the pleiotropic effects of statins in modulating immune responses are unclear (38). Reports of immune modulatory effects of statins have largely used supratherapeutic doses in vitro (39), whereas the statin type (lipophilic vs. hydrophilic) may have differential effects (40). The potential effects of statins are likely complex and context-dependent, given the reported downregulation of Th17 response in patients with NSTEMI undergoing coronary intervention with intensive statin therapy (41), yet the enhancement of immune checkpoint blockade immunotherapy (42). Another limitation is the characterization of Tf cells in patients only based on prior reports of ASCVD-associated alterations to adaptive immunity to P210 (5–7).

In conclusion, we have undertaken an investigation of the T cell response to the apoB-100 self-antigen P210 in patients with ASCVD and identified an impaired immune profile that parallels the reported low P210 IgG levels associated with higher risk of ASCVD (2–7), suggesting both cellular and humoral adaptive immune responses to P210 are impaired in ASCVD. Collectively, our findings reveal that intrinsic immunity against P210 appears to be protective given the impaired responses in patients with ASCVD. The observation that P210-PAM can partially reverse this immunological impairment suggests that the P210-specific immune deficit is modifiable. Consequently, these findings pave the way for novel immunotherapeutic interventions targeting P210. The study supports the next steps of developing an antigen-specific P210-based immune therapy targeting these pathways.

## Figures and Tables

**Figure 1.**
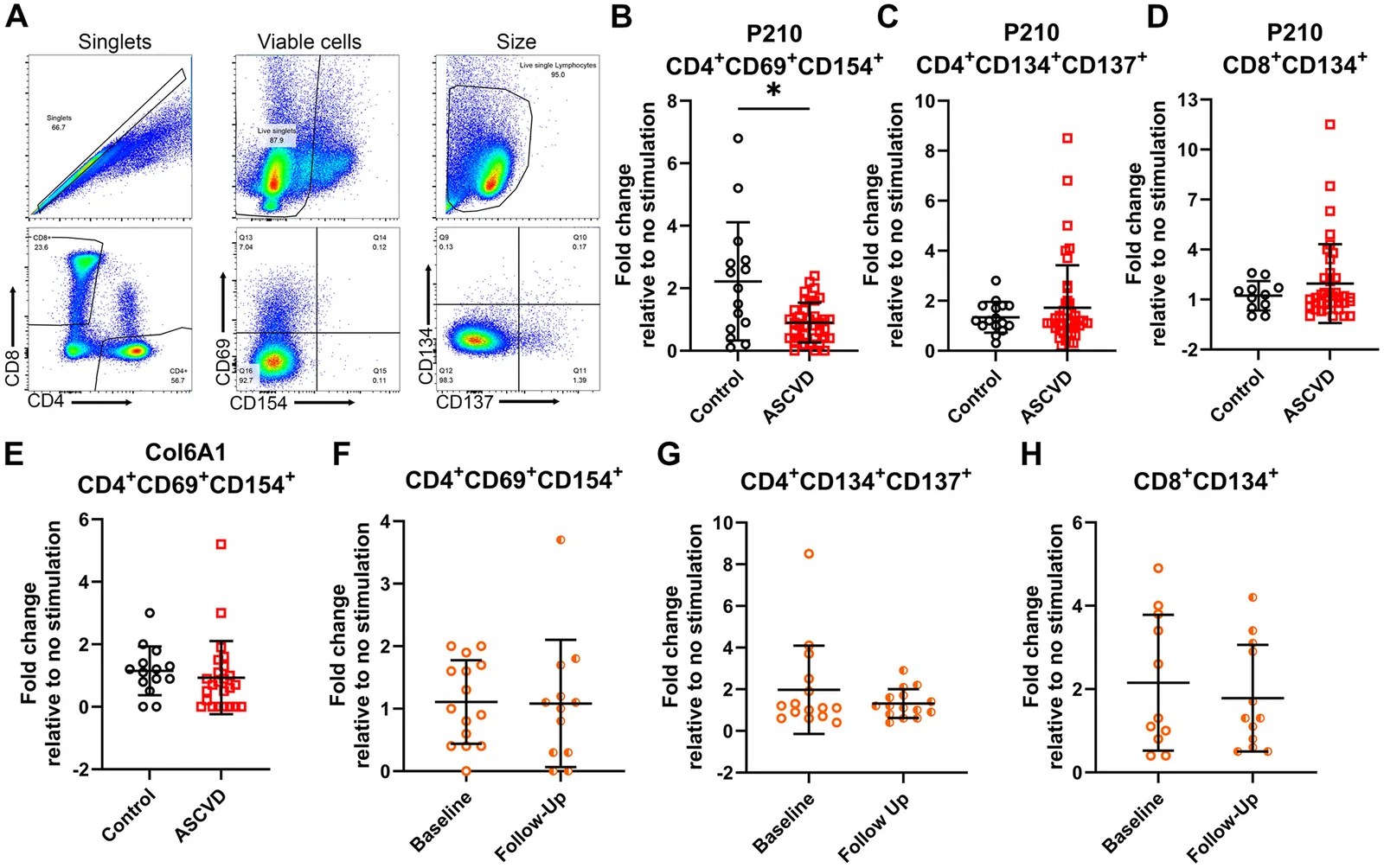
T cell response to P210 stimulation of PBMCs from patients with ASCVD. *A*: gating scheme for analysis of T cell activation in response to stimulation of healthy control and patient PBMCs with CD4^+^CD69^+^CD154^+^ T cells (control *n* = 16; ASCVD *n* = 37) (*B*), CD4^+^CD134^+^CD137^+^ T cells (control *n* = 16; ASCVD *n* = 43) (*C*), and CD8^+^CD134^+^ T cells (control *n* = 11; ASCVD *n* = 39) (*D*). *E*: T cell response to stimulation with unrelated Col6A1 peptide of healthy control (*n* = 14) and patient (*n* = 24) PBMCs. *B*: *P = 0.02, *t* test with Welch’s correction. Response to P210 in PBMCs of subgroup of patients with ASCVD (*n* = 15) with a history of MI or stroke at baseline and at approximately 1-year follow-up with CD4^+^CD69^+^CD154^+^ T cells (*F*), CD4^+^CD134^+^CD137^+^ T cells (*G*), and CD8^+^CD134^+^ T cells (*H*). ASCVD, atherosclerotic cardiovascular disease; PBMCs, peripheral blood mononuclear cells.

**Figure 2.**
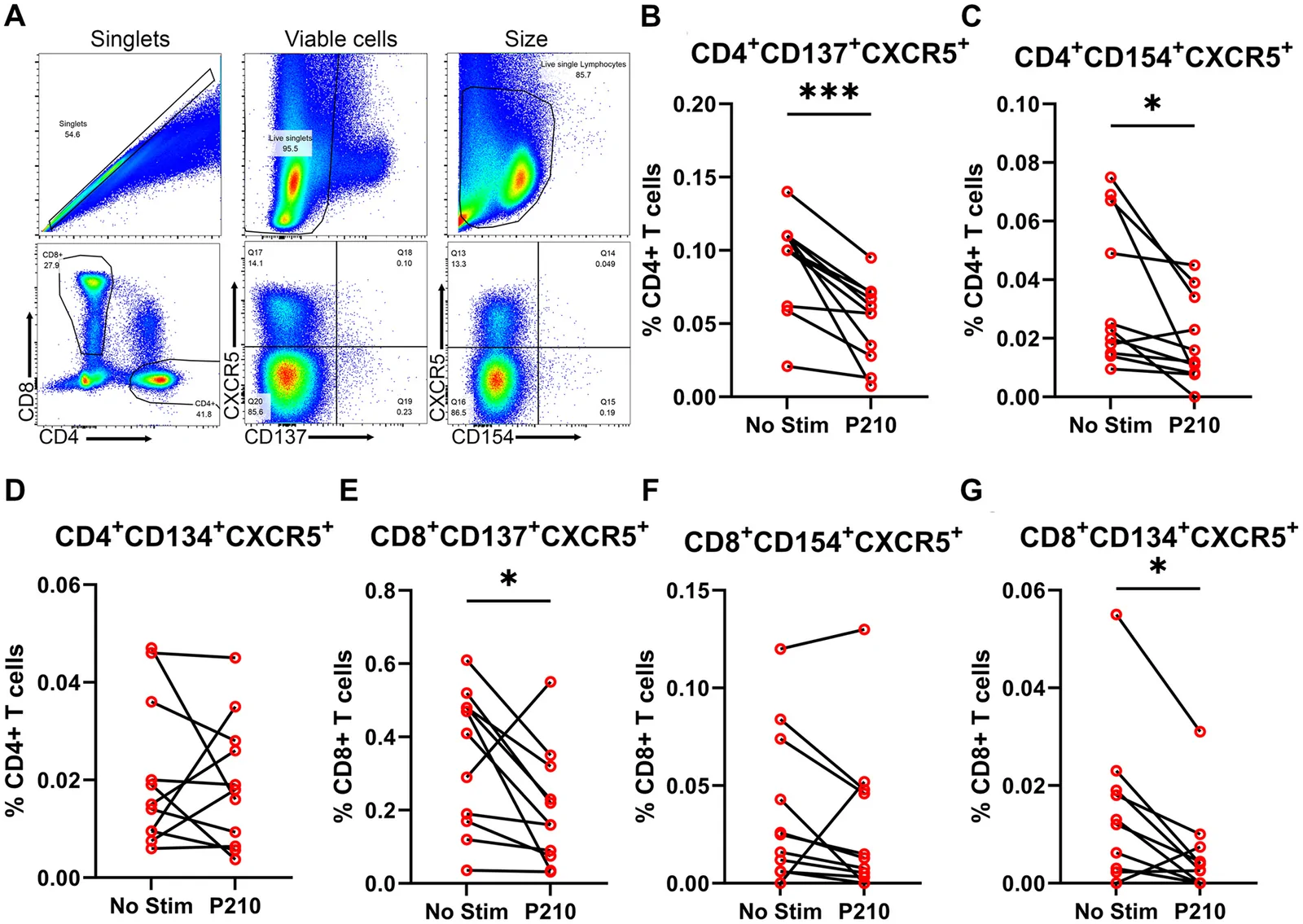
Reduced T follicular cells in PBMCs of patients with ACS in response to P210. *A*: gating scheme for analysis. CD4^+^CD137^+^CXCR5^+^ (*B*), CD4^+^CD154^+^CXCR5^+^ (*C*), and CD4^+^CD134^+^CXCR5^+^ Tfh cells (*D*) in response to P210 stimulation. ***P = 0.0006; *P = 0.02, paired *t* test. CD8^+^CD137^+^CXCR5^+^ (*E*), CD8^+^CD154^+^CXCR5^+^ (*F*), and CD8^+^CD134^+^CXCR5^+^ Tf-like cells (*G*) after P210 stimulation. *P = 0.03, paired *t* test (*E*); *P = 0.02, Wilcoxon paired rank test; *n* = 11 (*G*).

**Figure 3.**
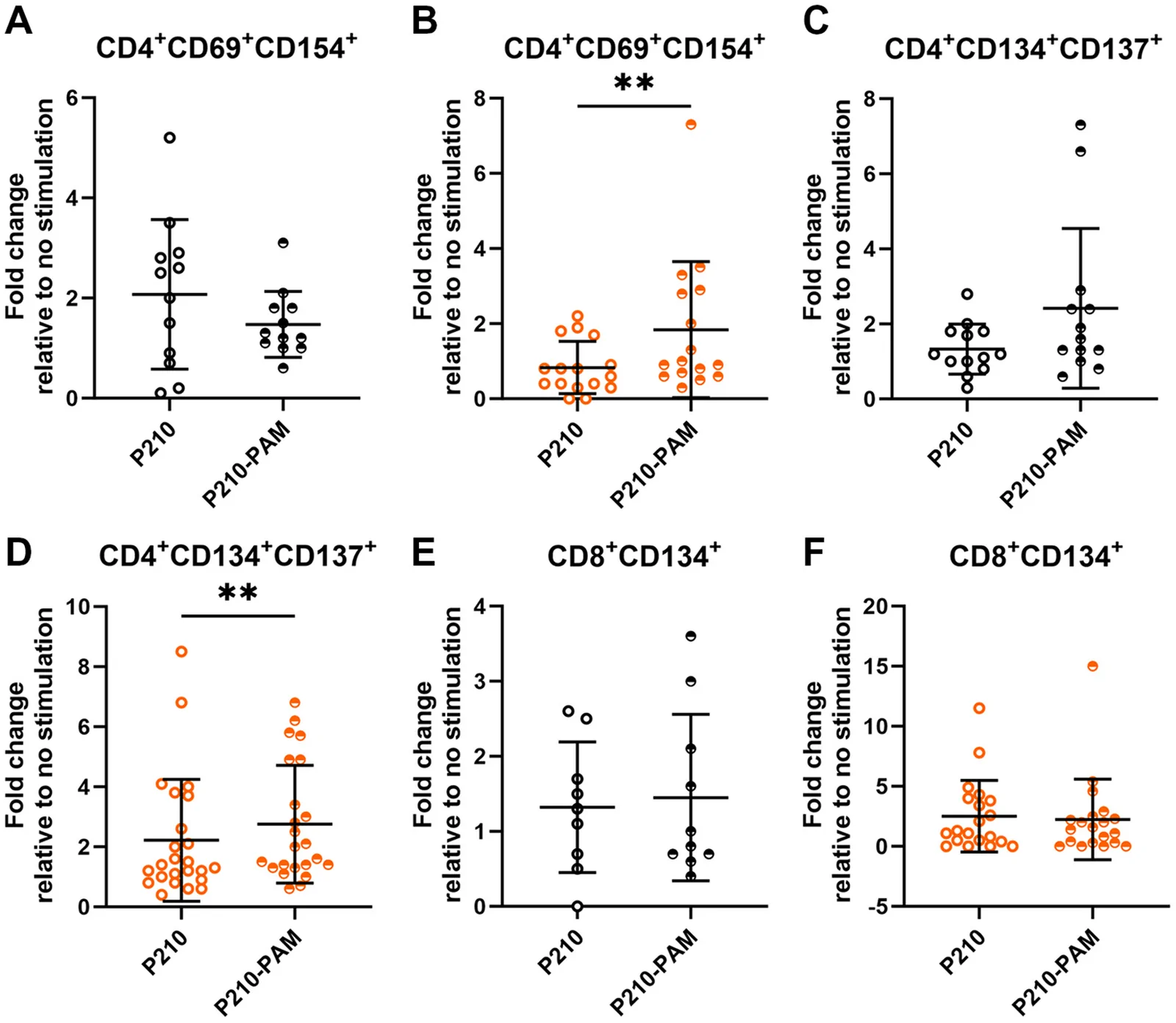
P210-PAM modifies T cell response to P210 in PBMCs of patients with ASCVD. CD4^+^CD69^+^CD154^+^ T cells in PBMCs of controls (*n* = 12) (*A*) and patients with ASCVD (*n* = 16) (*B*) stimulated with P210 or P210-PAM. CD4^+^CD134^+^CD137^+^ T cells in PBMCs of controls (*n* = 13) (*C*) and patients with ASCVD (*n* = 24) (*D*) stimulated with P210 or P210-PAM. CD8^+^CD134^+^ T cells in PBMCs of controls (*n* = 9) (*E*) and patients with ASCVD (*n* = 20) (*F*) stimulated with P210 or P210-PAM. **P = 0.006 (*B*); **P = 0.003, Wilcoxon test (*D*). ASCVD, atherosclerotic cardiovascular disease; PBMCs, peripheral blood mononuclear cells.

**Table 1.** ASCVD patient characteristics (n = 44)

Age, yr	59.0 ± 11.8
Female	14 (32)
Male	30 (68)
Non-ACS	
History of MI	14 (32)
History of stroke	13 (29)
PAD	6 (14)
ACS^a^	11 (25)
Use of statin	21 (48)
History of smoking	14 (32)
Diabetes	11 (25)
Hypertension	21 (48)

Categorical data presented as number of individuals (%); non-categorical data presented as means ± SD. ACS, acute coronary syndrome; ASCVD, atherosclerotic cardiovascular disease; MI, myocardial infarction; PAD, peripheral artery disease.

a ST elevation myocardial infarction and non-ST elevation myocardial infarction.

**Figure A1.**
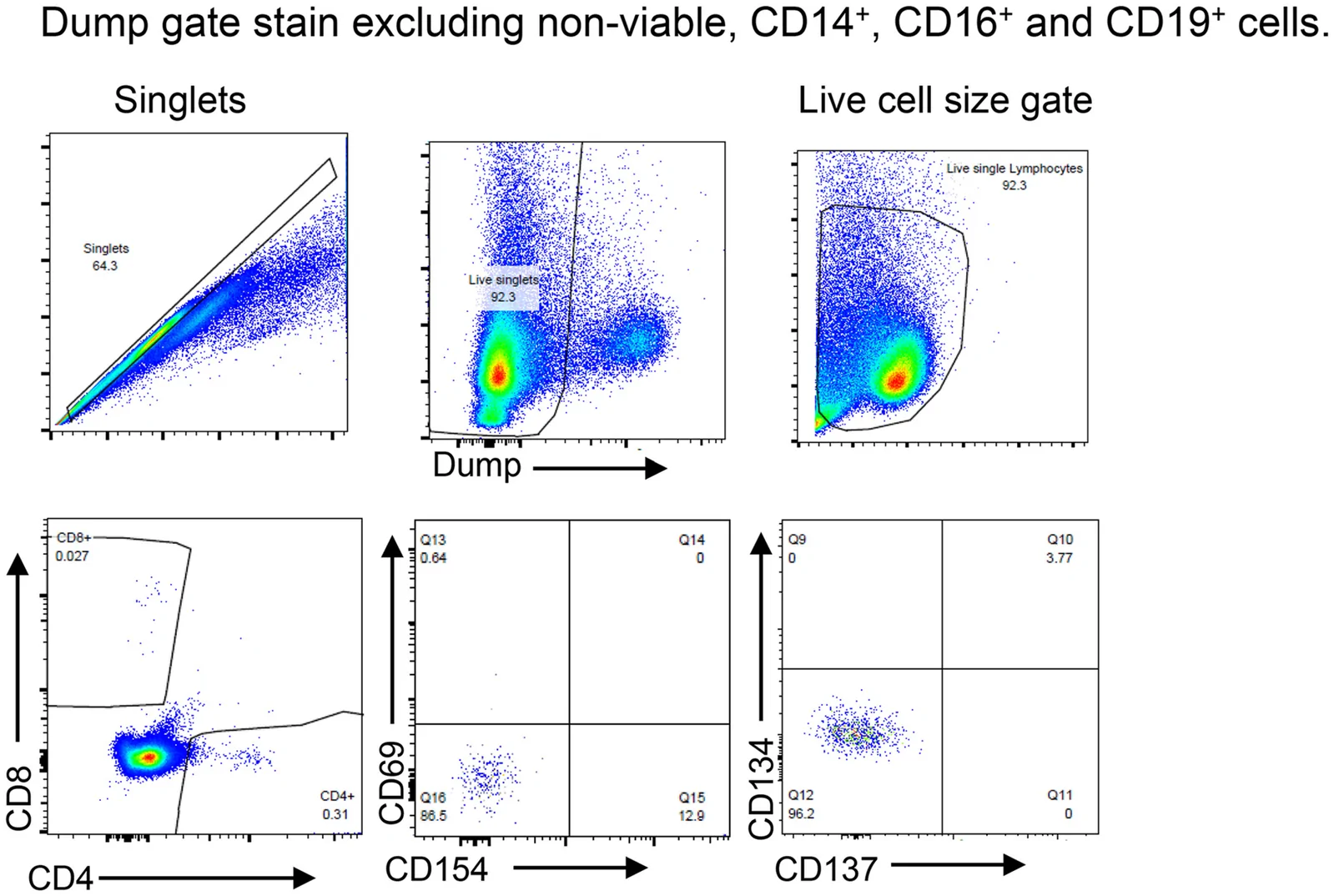
Gating scheme for flow cytometry analysis depicting exclusion of nonviable cells and CD14^+^, CD16^+^, and CD19^+^ cells in the dump gate.

## Data Availability

Data will be made available upon reasonable request.

## APPENDIX

Data presented below provide information regarding the antibody clones used for flow cytometry analysis (Table A1) and the gating strategy applied for identification and analysis of the cell populations of interest (Fig. A1).

**Table A1.** Antibody clones, catalog numbers, and sources used in the report

Marker	Clone	Cat. No.	Source
CD4	SK3	563550	BD Bioscience
CD8a	RPA-T8	48-0088	Invitrogen
CD25	M-A251	555431	BD Bioscience
CD69	FN50	555531	BD Bioscience
CD134	ACT35	563473	BD Bioscience
CD137	4B4 (4B4-1)	47-1379	Invitrogen
CD154	24-31	310838	BioLegend
CXCR5	J252DC	356903	BioLegend
CD14	61D3	69-0149	Invitrogen
CD16	CB16	69-0168	Invitrogen
CD19	HIB19	69-0199	Invitrogen

## References

[1] Fredrikson GN, Hedblad B, Berglund G, Alm R, Ares M, Cercek B, Chyu K-Y, Shah PK, Nilsson J. Identification of immune responses against aldehyde-modified peptide sequences in apoB associated with cardiovascular disease. Arterioscler Thromb Vasc Biol 23: 872–878, 2003. doi:10.1161/01.ATV.0000067935.02679.B0. 12649091

[2] Fagerberg B, Prahl Gullberg U, Alm R, Nilsson J, Fredrikson GN. Circulating autoantibodies against the apolipoprotein B-100 peptides p45 and p210 in relation to the occurrence of carotid plaques in 64-year-old women. PLoS One 10: e0120744, 2015. doi:10.1371/journal.pone.0120744. PMC4358991 25768285

[3] Asciutto G, Dias NV, Edsfeldt A, Alm R, Fredrikson GN, Gonçalves I, Nilsson J. Low levels of IgG autoantibodies against the apolipoprotein B antigen p210 increases the risk of cardiovascular death after carotid endarterectomy. Atherosclerosis 239: 289–294, 2015. doi:10.1016/j.atherosclerosis.2015.01.023. 25682025

[4] Svenungsson E, Engelbertsen D, Wigren M, Gustafsson JT, Gunnarsson I, Elvin K, Jensen-Urstad K, Fredrikson GN, Nilsson J. Decreased levels of autoantibodies against apolipoprotein B-100 antigens are associated with cardiovascular disease in systemic lupus erythematosus. Clin Exp Immunol 181: 417–426, 2015. doi:10.1111/cei.12651. PMC4557377 25959453

[5] Björkbacka H, Alm R, Persson M, Hedblad B, Nilsson J, Fredrikson GN. Low levels of apolipoprotein B-100 autoantibodies are associated with increased risk of coronary events. Arterioscler Thromb Vasc Biol 36: 765–771, 2016. doi:10.1161/ATVBAHA.115.306938. 26916732

[6] Sjogren P, Fredrikson GN, Samnegard A, Ericsson CG, Ohrvik J, Fisher RM, Nilsson J, Hamsten A. High plasma concentrations of autoantibodies against native peptide 210 of apoB-100 are related to less coronary atherosclerosis and lower risk of myocardial infarction. Eur Heart J 29: 2218–2226, 2008. doi:10.1093/eurheartj/ehn336. 18664466

[7] Imai M, Kawamura M, Kochi I, Matsuoka T, Kihara S, Yamamoto H. Anti-Apo B-100 autoantibody is a marker of unstable coronary plaque. J Atheroscler Thromb 28: 1025–1034, 2021. doi:10.5551/jat.58784. PMC8560848 33191364

[8] Hansson GK, Jonasson L. The discovery of cellular immunity in the atherosclerotic plaque. Arterioscler Thromb Vasc Biol 29: 1714–1717, 2009. doi:10.1161/ATVBAHA.108.179713. 19846836

[9] Ammirati E, Cianflone D, Vecchio V, Banfi M, Vermi AC, De MM, Grigore L, Pellegatta F, Pirillo A, Garlaschelli K, Manfredi AA, Catapano AL, Maseri A, Palini AG, Norata GD. Effector memory T cells are associated with atherosclerosis in humans and animal models. J Am Heart Assoc 1: 27–41, 2012. doi:10.1161/JAHA.111.000125. PMC3487313 23130116

[10] van Dijk RA, Duinisveld AJ, Schaapherder AF, Mulder-Stapel A, Hamming JF, Kuiper J, de Boer OJ, van der Wal AC, Kolodgie FD, Virmani R, Lindeman JH. A change in inflammatory footprint precedes plaque instability: a systematic evaluation of cellular aspects of the adaptive immune response in human atherosclerosis. J Am Heart Assoc 4: e001403, 2015. doi:10.1161/JAHA.114.001403. PMC4579929 25814626

[11] Fernandez DM, Rahman AH, Fernandez NF, Chudnovskiy A, Amir ED, Amadori L, Khan NS, Wong CK, Shamailova R, Hill CA, Wang Z, Remark R, Li JR, Pina C, Faries C, Awad AJ, Moss N, Bjorkegren JLM, Kim-Schulze S, Gnjatic S, Ma’ayan A, Mocco J, Faries P, Merad M, Giannarelli C. Single-cell immune landscape of human atherosclerotic plaques. Nat Med 25: 1576–1588, 2019. doi:10.1038/s41591-019-0590-4. PMC7318784 31591603

[12] Mayr M, Grainger D, Mayr U, Leroyer AS, Leseche G, Sidibe A, Herbin O, Yin X, Gomes A, Madhu B, Griffiths JR, Xu Q, Tedgui A, Boulanger CM. Proteomics, metabolomics, and immunomics on microparticles derived from human atherosclerotic plaques. Circ Cardiovasc Genet 2: 379–388, 2009. doi:10.1161/CIRCGENETICS.108.842849. 20031610

[13] Crotty S. Follicular helper CD4 T cells (TFH). Annu Rev Immunol 29: 621–663, 2011. doi:10.1146/annurev-immunol-031210-101400. 21314428

[14] Aloulou M, Fazilleau N. Regulation of B cell responses by distinct populations of CD4 T cells. Biomed J 42: 243–251, 2019. doi:10.1016/j.bj.2019.06.002. PMC6818157 31627866

[15] Elzein SM, Zimmerer JM, Han JL, Ringwald BA, Bumgardner GL. CXCR5^+^CD8^+^ T cells: a review of their antibody regulatory functions and clinical correlations. J Immunol 206: 2775–2783, 2021. doi:10.4049/jimmunol.2100082. PMC8486376 34602651

[16] Chyu KY, Zhao X, Dimayuga PC, Zhou J, Li X, Yano J, Lio WM, Chan LF, Kirzner J, Trinidad P, Cercek B, Shah PK. CD8 T cells mediate the athero-protective effect of immunization with an ApoB-100 peptide. PLoS One 7: e30780, 2012. doi:10.1371/journal.pone.0030780. PMC3276497 22347402

[17] Chyu K-Y, Zhao X, Zhou J, Dimayuga PC, Lio NW, Cercek B, Trac NT, Chung EJ, Shah PK. Immunization using ApoB-100 peptide-linked nanoparticles reduces atherosclerosis. JCI Insight 7: e149741, 2022. doi:10.1172/jci.insight.149741. PMC9220835 35536648

[18] Kelly CP, Murray JA, Leffler DA, Getts DR, Bledsoe AC, Smithson G, First MR, Morris A, Boyne M, Elhofy A, Wu T-T, Podojil JR, Miller SD; TAK-101 Study Group. TAK-101 nanoparticles induce gluten-specific tolerance in celiac disease: a randomized, double-blind, placebo-controlled study. Gastroenterology 161: 66–80.e8, 2021. doi:10.1053/j.gastro.2021.03.014. PMC9053078 33722583

[19] Mihailovic PM, Lio WM, Herscovici R, Chyu KY, Yano J, Zhao X, Zhou J, Zhou B, Freeman MR, Yang W, Shah PK, Cercek B, Dimayuga PC. Keratin 8 is a potential self-antigen in the coronary artery disease immunopeptidome: a translational approach. PLoS One 14: e0213025, 2019. doi:10.1371/journal.pone.0213025. PMC6392305 30811493

[20] Chernomordik F, Cercek B, Zhou J, Zhao X, Lio NWM, Chyu K-Y, Shah PK, Dimayuga PC. Impaired tolerance to the autoantigen LL-37 in acute coronary syndrome. Front Immunol 14: 1113904, 2023. doi:10.3389/fimmu.2023.1113904. PMC10083408 37051254

[21] Reiss S, Baxter AE, Cirelli KM, Dan JM, Morou A, Daigneault A, Brassard N, Silvestri G, Routy J-P, Havenar-Daughton C, Crotty S, Kaufmann DE. Comparative analysis of activation induced marker (AIM) assays for sensitive identification of antigen-specific CD4 T cells. PLoS One 12: e0186998, 2017. doi:10.1371/journal.pone.0186998. PMC5655442 29065175

[22] Lio WM, Cercek B, Yano J, Yang W, Ghermezi J, Zhao X, Zhou J, Zhou B, Freeman MR, Chyu KY, Shah PK, Dimayuga PC. Sex as a determinant of responses to a coronary artery disease self-antigen identified by immune-peptidomics. Front Immunol 11: 694, 2020. doi:10.3389/fimmu.2020.00694. PMC7187896 32373127

[23] Zaunders JJ, Munier ML, Seddiki N, Pett S, Ip S, Bailey M, Xu Y, Brown K, Dyer WB, Kim M, de Rose R, Kent SJ, Jiang L, Breit SN, Emery S, Cunningham AL, Cooper DA, Kelleher AD. High levels of human antigen-specific CD4^+^ T cells in peripheral blood revealed by stimulated coexpression of CD25 and CD134 (OX40). J Immunol 183: 2827–2836, 2009. doi:10.4049/jimmunol.0803548. 19635903

[24] Oh HS, Choi BK, Kim YH, Lee DG, Hwang S, Lee MJ, Park SH, Bae Y-S, Kwon BS. 4–1BB signaling enhances primary and secondary population expansion of CD8^+^ T cells by maximizing autocrine IL-2/IL-2 receptor signaling. PLoS One 10: e0126765, 2015. doi:10.1371/journal.pone.0126765. PMC4427336 25962156

[25] Elgueta R, Benson MJ, de Vries VC, Wasiuk A, Guo Y, Noelle RJ. Molecular mechanism and function of CD40/CD40L engagement in the immune system. Immunol Rev 229: 152–172, 2009. doi:10.1111/j.1600-065X.2009.00782.x. PMC3826168 19426221

[26] Alfaro C, Echeveste JI, Rodriguez-Ruiz ME, Solorzano JL, Perez-Gracia JL, Idoate MA, Lopez-Picazo JM, Sanchez-Paulete AR, Labiano S, Rouzaut A, Oñate C, Aznar A, Lozano MD, Melero I. Functional expression of CD137 (4–1BB) on T helper follicular cells. Oncoimmunology 4: e1054597, 2015. doi:10.1080/2162402X.2015.1054597. PMC4635945 26587331

[27] Dunér P, Mattisson IY, Fogelstrand P, Glise L, Ruiz S, Farina C, Borén J, Nilsson J, Bengtsson E. Antibodies against apoB100 peptide 210 inhibit atherosclerosis in apoE^−/−^ mice. Sci Rep 11: 9022, 2021. doi:10.1038/s41598-021-88430-1. PMC8079692 33907226

[28] Fredrikson GN, Andersson L, Soderberg I, Dimayuga P, Chyu KY, Shah PK, Nilsson J. Atheroprotective immunization with MDA-modified apo B-100 peptide sequences is associated with activation of Th2 specific antibody expression. Autoimmunity 38: 171–179, 2005. doi:10.1080/08916930500050525. 16040338

[29] Hermansson A, Johansson DK, Ketelhuth DF, Andersson J, Zhou X, Hansson GK. Immunotherapy with tolerogenic apolipoprotein B-100-loaded dendritic cells attenuates atherosclerosis in hypercholesterolemic mice. Circulation 123: 1083–1091, 2011. doi:10.1161/CIRCULATIONAHA.110.973222. 21357823

[30] Tse K, Gonen A, Sidney J, Ouyang H, Witztum JL, Sette A, Tse H, Ley K. Atheroprotective vaccination with MHC-II restricted peptides from ApoB-100. Front Immunol 4: 493, 2013. doi:10.3389/fimmu.2013.00493. PMC3873602 24416033

[31] Chyu KY, Zhao X, Reyes OS, Babbidge SM, Dimayuga PC, Yano J, Cercek B, Fredrikson GN, Nilsson J, Shah PK. Immunization using an Apo B-100 related epitope reduces atherosclerosis and plaque inflammation in hypercholesterolemic apo E (−/−) mice. Biochem Biophys Res Commun 338: 1982–1989, 2005. doi: 10.1016/j.bbrc.2005.10.141. 16288717

[32] Nguyen B, Tolia NH. Protein-based antigen presentation platforms for nanoparticle vaccines. NPJ Vaccines 6: 70, 2021. doi:10.1038/s41541-021-00330-7. PMC8119681 33986287

[33] Saleh M, El-Moghazy A, Elgohary AH, Saber WIA, Helmy YA. Revolutionizing nanovaccines: a new era of immunization. Vaccines (Basel) 13: 126, 2025. doi:10.3390/vaccines13020126. PMC11860605 40006673

[34] Stano A, Scott EA, Dane KY, Swartz MA, Hubbell JA. Tunable T cell immunity towards a protein antigen using polymersomes vs. solid-core nanoparticles. Biomaterials 34: 4339–4346, 2013. doi:10.1016/j.biomaterials.2013.02.024. 23478034

[35] Martin C, Côté-Cyr M, Nguyen PT, Archambault D, Bourgault S. Evaluation of cylindrical micelles assembled from amphiphilic β-peptides as antigen delivery nanostructures. Nanoscale Adv 7: 2979–2987, 2025. doi:10.1039/d5na00166h. PMC11960782 40177387

[36] Trent A, Ulery BD, Black MJ, Barrett JC, Liang S, Kostenko Y, David NA, Tirrell MV. Peptide amphiphile micelles self-adjuvant group A streptococcal vaccination. AAPS J 17: 380–388, 2015. doi:10.1208/s12248-014-9707-3. PMC4365084 25527256

[37] Zhang L, Al-Ammari A, Zhu D, Zhang H, Zhou P, Zhi X, Ding W, Li X, Yu Q, Gai Y, Ma X, Chen C, Zuo C, Zhang J, Zhu W, Sun D. A nanovaccine for immune activation and prophylactic protection of atherosclerosis in mouse models. Nat Commun 16: 2111, 2025. doi:10.1038/s41467-025-57467-5. PMC11873251 40025093

[38] Zhao TX, Mallat Z. Targeting the immune system in atherosclerosis: JACC state-of-the-art review. J Am Coll Cardiol 73: 1691–1706, 2019. doi:10.1016/j.jacc.2018.12.083. 30947923

[39] Blank N, Schiller M, Krienke S, Busse F, Schätz B, Ho AD, Kalden JR, Lorenz H-M. Atorvastatin inhibits T cell activation through 3-hydroxy-3-methylglutaryl coenzyme A reductase without decreasing cholesterol synthesis. J Immunol 179: 3613–3621, 2007. doi:10.4049/jimmunol.179.6.3613. 17785796

[40] Climent E, Benaiges D, Pedro-Botet J. Hydrophilic or lipophilic statins? Front Cardiovasc Med 8: 687585, 2021. doi:10.3389/fcvm.2021.687585. PMC8172607 34095267

[41] Ma X, Liu S, Li T, Yuan H. Intensive statin treatment ameliorate the Th17/Treg functional imbalance in patients with non-ST elevation acute coronary syndrome underwent percutaneous coronary intervention. Clin Cardiol 43: 379–385, 2020. doi:10.1002/clc.23326. PMC7144487 31872906

[42] Kristoff TJ, Evans S, Nayi P, Abousaud M, Goyal S, Liu Y, Shin D, Steuer CE, Saba NF, Schmitt NC. Statin drugs are associated with response to immune checkpoint blockade in recurrent/metastatic head and neck cancer. Cancer Med 14: e70718, 2025. doi:10.1002/cam4.70718. PMC11886884 40052634

